# Non-adherence to psychiatric medication in adults experiencing homelessness is associated with incurred concussions

**DOI:** 10.3389/fnhum.2022.958169

**Published:** 2022-07-28

**Authors:** Neal Rangu, Sumer G. Frank-Pearce, Adam C. Alexander, Emily T. Hébert, Chaelin Ra, Darla E. Kendzor, Michael S. Businelle

**Affiliations:** ^1^TSET Health Promotion Research Center, Stephenson Cancer Center, The University of Oklahoma Health Sciences Center, Oklahoma City, OK, United States; ^2^Department of Biostatistics and Epidemiology, Hudson College of Public Health, The University of Oklahoma Health Sciences Center, Oklahoma City, OK, United States; ^3^Department of Family and Preventive Medicine, College of Medicine, The University of Oklahoma Health Sciences Center, Oklahoma City, OK, United States; ^4^Department of Health Promotion and Behavioral Sciences, University of Texas Health Science Center at Houston, Houston, TX, United States

**Keywords:** medication adherence, mental health, traumatic brain injury, brain concussion, adults experiencing homelessness

## Abstract

This study investigated the relationship between concussions and medication adherence among 247 adults experiencing homelessness in Oklahoma City, Oklahoma, who were prescribed medication for a psychiatric disorder. Participants were asked whether they had “ever experienced a blow to the head that caused a concussion,” and medication adherence was measured by asking participants whether they had taken their psychiatric medication yesterday. The data were analyzed using univariate and multivariable logistic regressions. Results showed that more than half of the sample had a concussion history (61.9%), and homeless adults with a concussion history had higher odds of non-adherence to psychiatric medications compared with those who reported no concussion history [*OR* = 2.13 (*95% CI* = 1.08, 4.18)]. Findings suggest that medication non-adherence is associated with incurred concussions. Raising awareness among service providers of the relationship between traumatic brain injury and medication adherence may increase efforts to improve adherence in this underserved population.

## Introduction

Homelessness is a pervasive issue in the United States. Nearly 1.5 million Americans spend at least one night in an emergency shelter or transitional housing each year, and many of these individuals suffer from major psychiatric disorders (Solari et al., 2016; Ayano et al., 2019). For example, some of the most common psychiatric disorders in this population are schizophrenia spectrum disorders and major depression, and the prevalence of Schizophrenia (12.6 vs. 0.64%) and depression (12.6 vs. 6.7%) are substantially higher among adults experiencing homelessness than the general population (Gutwinski et al., 2021). Homeless adults are less likely to have access to psychiatric services than the general population (Kushel, 2001; Hwang et al., 2008) and even when treatment is provided, many homeless adults fail to adhere to the prescribed treatment (Coe et al., 2015; Hunter et al., 2015). Numerous studies have shown that around 25–35% of homeless adults prescribed medication will not stick to treatment. The primary reasons for non-adherence seem to be patient-related, such as running out of medication or poor self-management (Coe et al., 2015; Hunter et al., 2015). The management of major psychiatric disorders is profoundly affected by medication non-adherence (Farooq and Naeem, 2014) and non-adherence is associated with more hospitalizations, poor psychosocial outcomes and quality of life, and increased substance abuse and suicide risk (Novick et al., 2010; Ho et al., 2016). Therefore, it is essential to identify risk factors for medication non-adherence to improve mental health among homeless adults with psychiatric disorders.

Traumatic brain injuries (TBI), defined as an external force that disrupts or alters brain functioning, (Menon et al., 2010) are frequently reported by adults experiencing homelessness. A recent study found that the lifetime prevalence of TBI in homeless and marginally housed adults was 53.1%, and the lifetime prevalence of moderate or severe TBI was 22.5% (Kushel, 2001). TBIs impair cognition, particularly executive functioning, (Andersen et al., 2014; Karr et al., 2014) and a prospective study identified that even a mild TBI, also known as a concussion, is associated with significant structural changes to the brain that affect cognitive performance 1 year after injury (Zhou et al., 2013). Relatedly, numerous studies have shown that homeless adults with a history of TBI are more likely to have psychiatric disorders, use more emergency room services, report more unmet health care needs and memory concerns, and have more contact with the criminal justice system compared with those with no history of TBI (Hwang et al., 2008; Topolovec-Vranic et al., 2017; Stubbs et al., 2019).

No studies have determined whether TBIs are associated with medication adherence among homeless adults with psychiatric disorders despite TBIs being very common in this population (Hwang et al., 2008; Topolovec-Vranic et al., 2017). This study investigates the relationship between concussions and medication adherence among homeless adults who have been prescribed medication for a psychiatric disorder. We hypothesize that homeless adults with a concussion history will have a higher rate of non-adherence to psychiatric medication regimens compared with those who report no concussion history.

.

## Methods

This study is a secondary data analysis of a survey that was conducted at six homeless serving agencies, such as a day shelter or food bank, across Oklahoma City, Oklahoma, between July and August of 2016. Participants (*N* = 610) were recruited using flyers within each agency. Data from this survey were used previously to highlight the prevalence of modifiable health risk factors and investigated determinants of health among homeless adults living in Oklahoma City (Maness et al., 2019; Neisler et al., 2019; Taylor et al., 2019). Study eligibility requirements included: current homelessness, which was based on responses to questions related to the length of homelessness, place of current shelter, and reasons for homelessness. Participants who responded that they had been homeless for “0 months,” slept in their “personal apartment or house” last night, or reported they were “not currently homeless” were deemed not homeless (Neisler et al., 2018). Other criteria included: age ≥ 18 years, receiving services at a homeless shelter, (Neisler et al., 2018) and at least a 7th-grade reading level as indicated on the Rapid Estimate of Adult Literacy in Medicine Short Form (Arozullah et al., 2007). For this study, only participants who self-reported being prescribed medication for psychiatric disorders (i.e., Posttraumatic Stress Disorder, Major Depression, Bipolar Disorder, Schizophrenia or Schizoaffective Disorder, and other Anxiety Disorders) were included in the analyses (*n* = 247; 40.5% of the total sample). Each participant completed a series of measures on a tablet computer *via* software that read the questions aloud to the participant. Participants were instructed to talk with study staff if they had trouble answering a survey question. Participants were compensated with a $20 department store gift card for their participation.

### Measures

The independent variable, concussion history, was measured by asking participants whether they had “ever experienced a blow to the head that caused a concussion (Kay et al., 1993; Slaughter et al., 2003).” The dependent variable, medication adherence, was measured by asking homeless adults prescribed medication for Posttraumatic Stress Disorder, Major Depression, Bipolar Disorder, Schizophrenia or Schizoaffective Disorder, or other Anxiety Disorders whether they took their medication yesterday. Homeless adults often have more than one psychiatric disorder and may take numerous medications (Fazel et al., 2014). Therefore, we created a variable, which was included as a covariate in multivariable analyses to account for adults who were prescribed more than one medication for psychiatric disorders. Other covariates included self-reported race, sex, age, education, and insurance status.

### Analyses

Descriptive statistics were generated for independent and dependent variables and covariates. Next, univariate and multivariable logistic regressions were conducted to explore the associations between concussion history and medication adherence among homeless adults who were prescribed medication for a psychiatric disorder. These analyses were completed in SAS 9.4 (SAS, 2013).

## Result

The sample of participants (*n* = 247) included in this study was primarily White (59.3%) and male (57.9%), with an average age of 44.2 years (*SD* = 11.8). Most homeless adults had education equal to or less than a high school diploma (66%), and most of the sample did not have health insurance (65.1%). More than half of the sample had a concussion history (61.9%), and many self-reported a history of a psychiatric disorder, including 29.2% with Schizophrenia or Schizoaffective Disorder, 52.2% with Posttraumatic Stress Disorder, 67.2% with an Anxiety Disorder, 91.5% with Major Depression, 47% with Bipolar Disorder, and 45.8% with a Substance Use Disorder (excluding tobacco use). There were no significant differences in self-reported psychiatric disorders among those with and without a concussion history (see Table 1 for additional details).

**Table 1:** Participant characteristics (*N* = 247).

**Variable**	**Concussion history**		***p***
	**No (*****n*** **= 94)**	**Yes (*****n*** **= 153)**	
Race			
White	51 (54.3%)	93 (62.4%)	0.21
Non-white^a^	43 (45.7%)	56 (37.6%)	
Sex			
Male	50 (53.2%)	93 (60.8%)	0.24
Female	44 (46.8%)	60 (39.2%)	
Age	41.14 (SD = 11.7)	46.1 (SD = 11.5)	<0.01
Education			
High school degree or less	59 (62.8%)	104 (68.0%)	0.40
Some college or more	35 (37.2%)	49 (32.0%)	
Mental health history			
Schizophrenia or schizoaffective disorder	29 (30.9%)	43 (28.1%)	0.64
Posttraumatic stress disorder	42 (44.7%)	87 (56.9%)	0.06
Anxiety disorder	57 (60.6%)	109 (71.2%)	0.08
Major depressive disorder	86 (91.5%)	140 (91.5%)	0.99
Bipolar disorder	39 (41.5%)	77 (50.3%)	0.18
Substance use disorder	39 (41.5%)	74 (48.4%)	0.29
Number of prescribed psychiatric medications	2.4 (SD = 1.1)	2.6 (SD = 1.2)	0.26
Health insurance status			
No insurance	64 (68.1%)	97 (63.4%)	0.45
Any insurance	30 (31.9%)	56 (36.6%)	

SD, Standard deviation.

a African Americans (n = 42), Native Hawaiian or Other Pacific Islander (n = 2), American Indian/Alaska Native (n = 25), more than one race (n = 28), and Other (n = 2).

All variables have <1% missing data.

Overall, homeless adults with a concussion history had more than two times the odds of being non-adherent to their prescribed psychiatric medication (i.e., failing to take psychiatric medication on the previous day) compared with adults with no concussion history [*OR* = 2.13 (*95% CI* = 1.08, 4.18)]. As shown in Table 2, homeless adults with a concussion history had six times the odds of non-adherence (i.e., failing to take psychiatric medication on the prior day) to their prescribed medication for Schizophrenia or Schizoaffective Disorder compared with those with no concussion history [*OR* = 6.45 (*95% CI* = 1.23, 33.83)]. Similarly, homeless adults with a concussion history had more than quadruple the odds of non-adherence to their prescribed medication for Posttraumatic Stress Disorder compared with those with no concussion history [*OR* = 4.14 (*95% CI* = 1.18, 14.49)]. Among homeless adults prescribed medication for an Anxiety Disorder, the odds of non-adherence to prescribed medication were three times higher among homeless adults with a concussion compared to those with no concussion history [*OR* = 3.06 (*95% CI* = 1.41, 6.65)]. There was no significant difference in the odds of non-adherence among homeless adults with and without a concussion history for those prescribed medication for depression [*OR* = 2.00 (*95% CI* = 0.90, 4.39)] or bipolar disorder [*OR* = 2.92 (*95% CI* = 0.87, 9.76)].

**Table 2:** Association between concussion history and medication non-adherence among homeless adults prescribed medication for a psychiatric disorder (0 = adherent, 1 = not adherent).

**Dependent variable**	**Concussion history** **(No vs. Yes)** ^a^	
	**OR (95% CI)**	**aOR (95% CI)** ^b^
Any psychiatric medication	1.64 (0.90, 2.99)	2.13 (1.08, 4.18)
Schizophrenia or schizoaffective disorder medication	3.69 (1.14, 12.00)	6.45 (1.23, 33.83)
Posttraumatic stress disorder medication	3.89 (1.19, 12.74)	4.14 (1.18, 14.49)
Anxiety disorder medication	2.34 (1.18, 4.64)	3.06 (1.41, 6.65)
Major depressive disorder medication	2.04 (0.98, 4.24)	2.00 (0.90, 4.39)
Bipolar disorder medication	2.51 (0.84, 7.48)	2.92 (0.87, 9.76)

*Adherence to psychiatric medication is based on the question, “Did you take your X (Schizophrenia or Schizoaffective disorder, Posttraumatic Stress Disorder, Anxiety, Bipolar, or Major Depression disorder) medication yesterday.” All analyses have <5% missing data*.

*OR, Odds ratio, CI, Confidence Interval*.

a *No concussion history is the reference group for analyses*.

b *Multivariable models were adjusted for race, sex, education, age, total psychiatric medication prescribed, and insurance status*.

## Discussion

This study aimed to investigate the relation between concussions and medication adherence among homeless adults prescribed medication for a psychiatric disorder. The results were mostly consistent with our hypothesis; homeless adults with a concussion history had higher odds of non-adherence to psychiatric medication related to Schizophrenia or Schizoaffective Disorder, Posttraumatic Stress Disorder, and anxiety disorder than those without a concussion history. Though the effect sizes were large for Bipolar and Major Depressive Disorder, we failed to detect a significant association. Overall, homeless adults with a concussion history have a higher risk than those without a history of non-adherence to psychiatric medications, though adherence varies by psychological diagnosis.

Overall, medication adherence is very low among adults experiencing homelessness, (Unni et al., 2014) and the high prevalence of TBIs in this population may further interfere with adherence. Homeless adults often self-report that poor self-management skills (35%) and forgetfulness (12%) are significant reasons for non-adherence to psychiatric medication and other treatments for psychiatric disorders (Coe et al., 2015). Poor self-management skills and memory loss may indicate deficits in cognitive performance and executive functioning, (Coe et al., 2015; Stone et al., 2019) which are strongly correlated with TBIs (Andersen et al., 2014). It may be sensible for clinicians to screen for TBI history before providing psychiatric medication to homeless adults with psychiatric disorders. Relatedly, the use of telemedicine, and adherence technologies, such as text messaging programs, adherence apps, and smart pill bottles, should also be considered as ways to boost medication adherence (Ennis et al., 2015; Thakkar et al., 2016; Steinkamp et al., 2019).

Results from this study should be cautiously interpreted because there are some notable limitations. First, the cross-sectional design of this study limits conclusions about causation. It is plausible that non-adherence to psychiatric medication could increase the odds of a concussion by influencing risk-taking behavior (Basit et al., 2020). More importantly, certain psychological disorders and health conditions, such as drug use disorders, Schizophrenia, and epilepsy, are associated with medication non-adherence and can affect cognitive performance, regardless of TBI or concussion history (Reddy et al., 2014). Further, these psychological and physical health conditions may also contribute to an adult experiencing a concussion. For example, a person with severe drug use disorder may experience a TBI during a bout of intoxication (Haddad et al., 2014). Future research needs to consider using prospective data to replicate the observed associations to reduce the bias inherent in cross-sectional design studies.

Second, this study used a one-item self-report measure of concussion and psychiatric disorders, and the self-reported history of Schizophrenia and schizoaffective disorders were measured in the same question. Future studies should consider measuring these variables using medical records or validated symptom inventories to obtain more objective and valid information about the history of TBI exposure and psychiatric disorders. Further, although these disorders share some characteristics (Weil et al., 2018) future studies should consider assessing Schizophrenia and schizoaffective disorders separately. Third, concussion severity was not assessed in this study, which may indicate greater cognitive impairment. Fourth, this study focuses only on medication adherence with psychiatric medication, and the findings may not generalize to homeless adults taking medication for physical disorders, such as epilepsy. Fifth, we did not use a validated measure for medication adherence, nor did we ask adults whether they had access to their psychiatric medication, a common factor associated with non-adherence to medication use within this population (Hartman et al., 2019; Richler et al., 2019).

Sixth, although some individuals may have recovered from their psychiatric disorder and no longer require psychiatric medication, some disorders, such as Schizophrenia, require long-term treatment (Moilanen et al., 2016) and the lack of a prescription for medication could also indicate poor medication adherence. However, we did not have enough information to identify these participants and therefore chose to limit the analyses to participants with an active prescription for psychiatric medication. Seventh, this sample was recruited solely from working with agencies that serve homeless adults. A small segment of adults who experience homelessness do not routinely seek services and may remain transient. This population may also have more difficulty adhering to medication than the adults actively seeking services. Finally, future studies should recruit a larger sample of homeless adults with psychiatric disorders. Though the observed effect sizes for medication adherence related to Major Depressive Disorder and Bipolar Disorder were large, more participants may be needed to detect a significant difference in the odds of medication adherence for these disorders among homeless adults with and without a concussion history.

In summary, the lifetime prevalence of TBI in homeless and marginally housed adults is 53.1%, which suggests that TBIs are a serious and common health problem affecting this underserved population (Stubbs et al., 2019). This study provides evidence that TBI may also be associated with health behavior, particularly medication adherence for psychiatric disorders. Medication adherence affects the management of psychiatric disorders (Farooq and Naeem, 2014) and TBIs may interfere with treating psychiatric disorders in homeless populations. Health care providers should consider assessing TBI history in this population when prescribing psychiatric medications. Early identification of TBI history could help improve medication adherence in the homeless population by allowing providers to provide resources and teach practical strategies for adherence when prescribing medications. For example, providers can give patients daily pill organizers, help patients set phone-based medication reminder alarms, automate prescription refills at their preferred pharmacy and/or automatically mail prescriptions to shelter caseworkers. There is also a need to educate neurologists and neurosurgeons about the prevalence and severity of TBIs within this population so that these medical professionals can be ready to address TBI history during emergency room visits. Overall, more research is needed to develop and evaluate interventions to improve medication adherence in this understudied and underserved population. Increasing medication adherence will improve mental health and may improve various health and psychosocial outcomes among adults experiencing homelessness (Farooq and Naeem, 2014).

## Data Availability Statement

The raw data supporting the conclusions of this article will be made available by the authors, without undue reservation.

## Ethics Statement

The studies involving human participants were reviewed and approved by Institutional Review Board of the University of Oklahoma Health Sciences Center. The patients/participants provided their written informed consent to participate in this study.

## Author contributions

MB designed the parent study. NR and AA formulated the research questions, hypotheses, and prepared the first draft of the manuscript. SF-P conducted the secondary data analyses for this study. All authors revised the first draft and approved the final manuscript.

## Funding

This research and preparation of this manuscript were supported by the Oklahoma Tobacco Settlement Endowment Trust (092-016-0002). Funding for this project was also supported by the American Cancer Society Grant MRSGT-12-114-01-CPPB. Manuscript preparation was additionally supported by the National Institute on Minority Health and Health Disparities [Grant Number 1K01MD015295-01A1] and National Cancer Institute Cancer Center Support Grant P30CA225520 awarded to the Stephenson Cancer Center.

## Conflict of interest

The authors declare that the research was conducted in the absence of any commercial or financial relationships that could be construed as a potential conflict of interest.

## Publisher's note

All claims expressed in this article are solely those of the authors and do not necessarily represent those of their affiliated organizations, or those of the publisher, the editors and the reviewers. Any product that may be evaluated in this article, or claim that may be made by its manufacturer, is not guaranteed or endorsed by the publisher.
